# Analysis of Chemical Composition, Antioxidant Capacity, Acute Toxicity, and Antinociceptive Properties of Aqueous Extract of Origanum Majorana L.

**DOI:** 10.1002/cbdv.202401580

**Published:** 2024-11-18

**Authors:** My Ahmed El Amiri, Hamid Kabdy, Abdelfatah Aitbaba, Jawad Laadraoui, Rachida Aboufatima, Loubna El Yazouli, Baslam Abdelmounaim, Soad Moubtakir, Stefania Garzoli, Chait Abderrahman

**Affiliations:** ^1^ Laboratory of biochemistry Faculty of medicine and pharmacy University Cadi Ayyad Marrakech Morocco; ^2^ Laboratory of Pharmacology Neurobiology and Behavior Faculty of Sciences Semlalia University Cadi Ayyad Marrakech Morocco; ^3^ Laboratory of Physiopathology Genetic Molecular and Biotechnology Faculty of Sciences Aïn Chock Hassan II University Casablanca Morocco; ^4^ Laboratory of Biological Engineering Faculty of Sciences and Technology Sultan Moulay Slimane University Beni Mellal 23000 Morocco; ^5^ Department of Chemistry and Technologies of Drug Sapienza University P. le Aldo Moro 500185 Rome Italy

**Keywords:** *Origanum majorana* L, Phytochemical composition, Antioxidant, Toxicity, Antinociceptive

## Abstract

Pharmacological evaluation showed that AEOM significantly reduced pain in an animal model, suggesting potential analgesic properties. Acute toxicity studies indicated no adverse effects on kidney and liver function or blood parameters at doses up to 800 mg/kg. The analgesic effect is likely mediated by flavonoids in the extract, which may inhibit pain pathways. These findings suggest that O. majorana has promising therapeutic applications, particularly as a natural analgesic agent.

## Graphical abstract

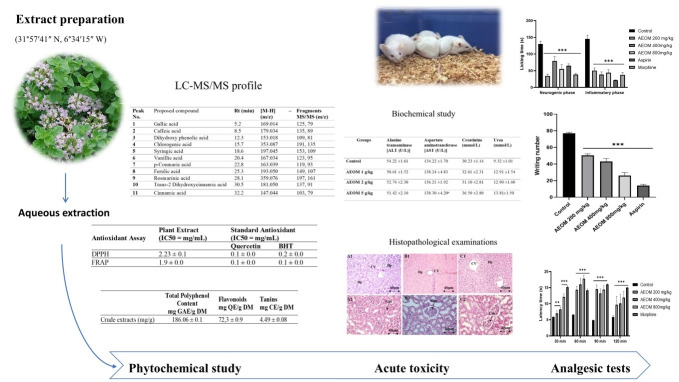

## Introduction

In recent decades, herbal remedies have gained global recognition due to their effectiveness, affordability, and limited side effects in the treatment of diseases. Natural products, particularly those derived from plants, play a key role as important sources of raw materials. These resources have led to the discovery of numerous bioactive molecules, offering various benefits in the fields of nutrition, cosmetics, and pharmaceuticals.^[1]^

Morocco, known for its rich biodiversity, boasts an impressive variety of plant species. Among the 4,200 species and subspecies of vascular plants in the country, 800 are endemic.^[2]^ This diversity underscores the value of Moroccan flora, which has long been exploited for medicinal purposes. As a result, research into medicinal plants has intensified, exploring their potential as sources of alternative remedies.^[3]^ Among these, *Origanum majorana* L., known as sweet marjoram, is a medicinal plant of the Lamiaceae family, widely used in traditional medicine for its various therapeutic properties.[^4^, ^5^] This plant, primarily distributed in Mediterranean regions, is rich in phytochemicals such as thymol, carvacrol, and various flavonoids, which explain its numerous biological properties.[^6^, ^7^, ^8^, ^9^]

*Origanum majorana*, commonly used in traditional medicine, has therapeutic applications depending on the plant parts used. In Moroccan traditional medicine, the leaves are primarily utilized for their anti‐cooling, antipyretic, and antihypertensive properties, as well as for treating allergies, fever, flu, and respiratory infections[^10^, ^11^, ^16^] Additionally, the leaves and stems are effective against rheumatism,[^11^, ^13^] stomach pain, headache, cough, and insomnia.^[14]^ The plant is also used in infusion form for calming, antispasmodic effects, and treating colds, fever, and headaches[^15^, ^16^]

The extracts of *O. majorana* exhibit notable biological activities, including antibacterial properties against various pathogenic bacteria and antifungal effects against pathogenic fungi.^[17]^ Furthermore, *O. majorana* possesses antiparasitic, antidiabetic, anti‐inflammatory, analgesic, antipyretic, hepatoprotective, antimutagenic, and gastrointestinal properties.[^18^, ^19^, ^20^, ^21^, ^22^] In this context, our study aimed to experimentally evaluate the traditional uses of the aqueous extract of *O. majorana* on animal models. We have examined the chemical composition of this extract, its antioxidant properties, acute toxicity, and antinociceptive effects.

## Results and Discussion

### HPLC Ms/Ms Analysis

Chemical analysis of the aqueous extract of *O. majorana*, conducted via LC–MS/MS, has enabled the identification of a variety of phenolic compounds, as detailed in Table 1. Among them, gallic acid, caffeic acid, and chlorogenic acid stand out for their pronounced bioactive properties. Additionally, other minor compounds have been identified in this variety originating from the Azilal region.

**Table 1:** Annotated compounds from *O. majorana* aqueous extract using LC–MS/MS.

Peak No.	Proposed compound	Rt (min)	[M−H] – (m/z)	Fragments MS/MS (m/z)
1	Gallic acid	5.2	169.014	125, 79
2	Caffeic acid	8.5	179.034	135, 89
3	Dihydroxy phenolic acid	12.3	153.018	109, 81
4	Chlorogenic acid	15.7	353.087	191, 135
5	Syringic acid	18.6	197.045	153, 109
6	Vanillic acid	20.4	167.034	123, 95
7	p‐Coumaric acid	22.8	163.039	119, 93
8	Ferulic acid	25.3	193.050	149, 107
9	Rosmarinic acid	28.1	359.076	197, 161
10	Trans‐2 Dihydroxycinnamic acid	30.5	181.050	137, 91
11	Cinnamic acid	32.2	147.044	103, 79

### Phytochemical Study

Our results showed significant and remarkable levels of total polyphenols (186.06±0.1 mg GAE/g), flavonoids (72.3±0.9 mg QE/g), and condensed tannins (4.49±0.08 mg CE/g) in AEOM leaves. (Table 2).

**Table 2:** Mean values of total polyphenol, flavonoids, and tannin contents in the extract.

	Total Polyphenol Content mg GAE/g DM	Flavonoids mg QE/g DM	Tanins mg CE/g DM
Crude extracts (mg/g)	186.06±0.1	72.3±0.9	4.49±0.08

GAE: gallic acid equivalent, QE: Qercetin equivalent, CE: Catechin equivalent, DM: dry matter.

### Antioxidant Activity

AEOM exhibited antioxidant activity similar to that of a standard antioxidant, as evaluated through both the DPPH scavenging assay and the reducing power assay. The calculated IC_50_ value was 2.23±0.03 mg/mL in the DPPH assay, indicating its capability to convert the stable purple‐colored DPPH radical into the yellow‐colored DPPH‐H form (Table 3). Additionally, AEOM demonstrated reducing power, with an IC_50_ value of 1.9±0.01 mg/mL

**Table 3:** The antioxidant activity of the AEOM by DPPH and FRAP.

Antioxidant Assay	Plant Extract (IC_50_=mg/mL)	Standard Antioxidant (IC_50_=mg/mL)	
		Quercetin	BHT
DPPH	2.23±0.1	0.1±0.0	0.2±0.0
FRAP	1.9±0.0	0.1±0.0	0.1±0.0

Data are mean±SEM.

### Estimation of LD_50_

The oral administration of single doses (1000, 2000, and 5000 mg/kg body weight) of AEOM did not lead to any mortality among the treated animals during the fourteen‐day observation period. No signs of toxicity were observed, and no significant alterations in body weight or relative organ weight were found among the treated animals. These results indicated that the oral lethal dose 50 (LD_50_) of AEOM exceeded 5000 mg/kg body weight in mice.

### Biochemical and Histological Analyses

**Figure 1:**
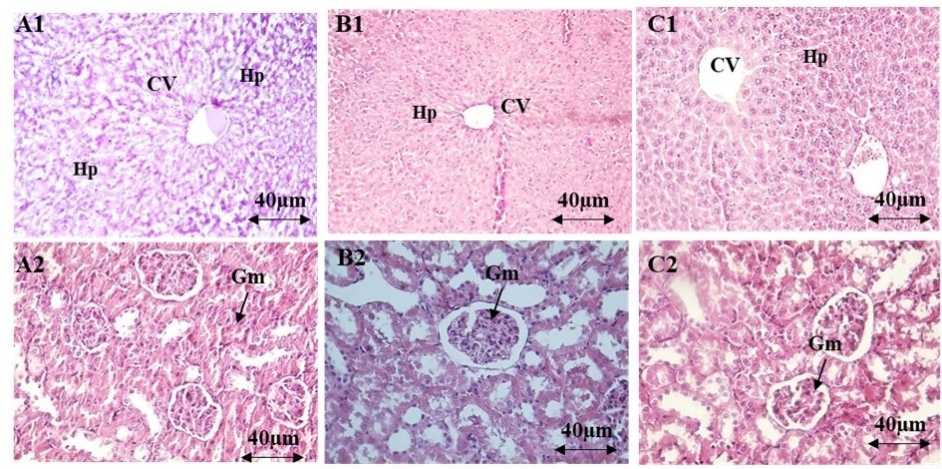
Histopathological examinations of organs (liver, kidneys) in acute toxicity. Liver tissue: Control (A1), AEOM 2 g/kg (B1), and AEOM 5 g/kg (C1). Kidney tissue: Control (A2), AECH 2 g/kg (B2), and AEOM 5 g/kg (C2). Sections were stained with hematoxylin and eosin (H&E) and viewed at ×200 magnification.

The administration of AEOM did not affect the levels of biochemical parameters (urea, creatinine, ASAT, ALAT) at any dose (Table 4). Microscopic examination of the kidney and liver tissues showed a normal appearance similar to that of the control group, indicating that no damaging changes or morphological disturbances were caused by the oral administration of AEOM (Figure 1).

**Table 4:** Biochemical parameters in the serum of mice treated with AEOM.

Groups	Alanine transaminase [ALT (U/L)]	Aspartate aminotransferase [AST (U/L)]	Creatinine (mmol/L)	Urea (mmol/L)
Control	54.22±1.61	134.22±1.70	30.23±1.14	9.32±1.01
AEOM 1 g/kg	50.61±1.52	138.24±4.83	32.61±2.31	12.91±1.54
AEOM 2 g/kg	52.74±2.30	136.21±1.92	31.10±2.81	12.90±1.60
AEOM 5 g/kg	51.42±2.10	138.30±4.20^[a]^	36.50±2.80	13.81±1.50

Data are expressed as mean±SEM, [a] indicate p<0.01 compared with saline‐treated controls.

### Antinociceptive Activity

#### Hot Plate Test

**Figure 2:**
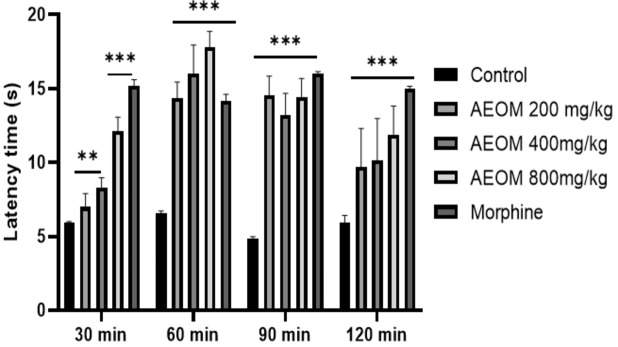
Effect of AEOM on latency time in the hot plate test with time course (n=6 per group). AEOM (200, 400, and 1000 mg/kg) and morphine (10 mg/kg, i. p.) were administered 30 minutes before the test. The results are presented as mean±SEM. Statistical analyses were performed using one‐way ANOVA followed by the post hoc multiple comparisons test. **p<0.01, *** *p*<0.001.

The results of the analgesic effects of the plant extract and the standard drug are illustrated in Figure 2. The antinociceptive activity results reveal that AEOM significantly increased (*p*<0.001) time latency at 30, 60, and 90 minutes compared to the control group. Similarly, the standard drug (morphine 10 mg/kg) significantly prolonged the time intervals in all cases compared to the control group.

#### Abdominal Writhing

**Figure 3:**
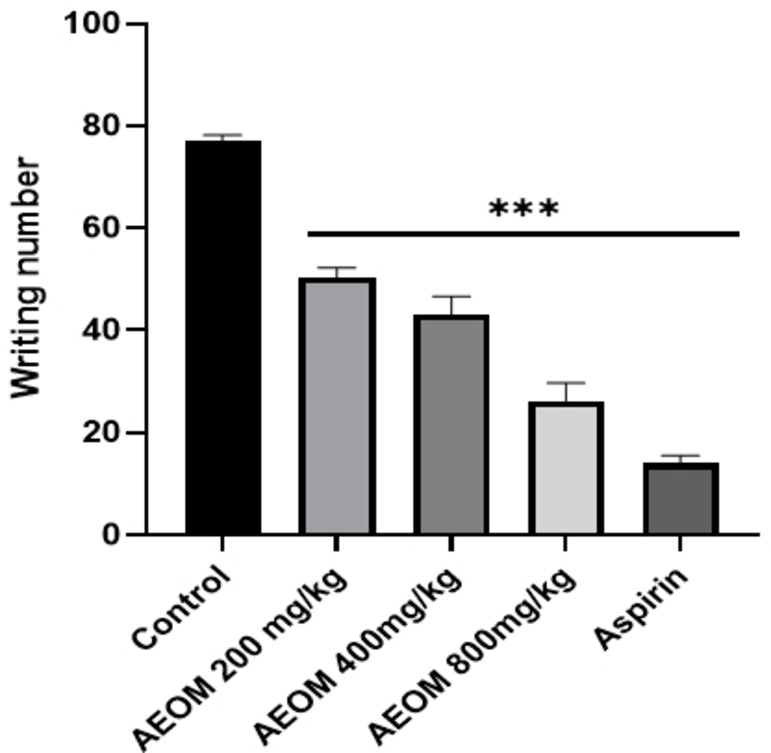
Effect of AEOM on the number of writhing responses in the writhing test. (n=6 per group). AEOM (200, 400, and 800 mg/kg), Acetylsalicylic acid (ASA); 200 mg/kg, i. p.) were administered 30 minutes before the test. The results are presented as mean±SEM. Statistical analyses were performed using one‐way ANOVA followed by the post hoc multiple comparisons test. *** vs control, *** *p*<0.001.

The application of AEOM at all dosage levels resulted in a significant reduction in the number of abdominal constrictions compared to the control group (Figure 3). This effect was more pronounced at higher doses (*p*<0.01) and was comparable to the effects observed in the morphine and aspirin groups, which served as positive controls.

#### Formalin Test

**Figure 4:**
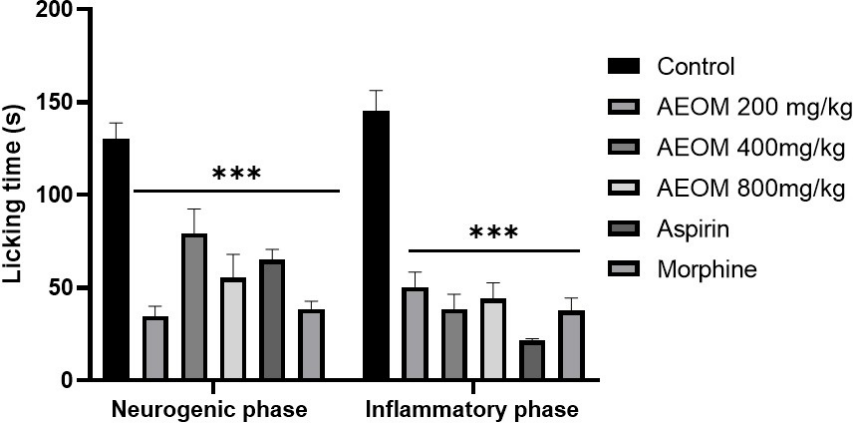
Effect of AEOM during the early and late phases in the formalin test (n=6). AEOM (200, 400, and 800 mg/kg), morphine (10 mg/kg, i. p.), and ASA (200 mg/kg, i. p.) were administered 30 minutes before the test. The results are presented as mean±SEM. The results are presented as mean±SEM. Statistical analyses were performed using one‐way ANOVA followed by the post hoc multiple comparisons test. ***vs control, *** *p*<0.001.

The formalin test results are shown in Figure 4. The extract exhibited notable analgesic effects by decreasing paw licking time during both the neurogenic (Phase 1) and inflammatory (Phase 2) phases, with the reduction being particularly evident in this last phase. Administration of AEOM at doses of 200, 400, and 800 mg/kg resulted in reduced paw licking time during both phases of the formalin trial. In comparison, the reference drug, aspirin, demonstrated greater efficacy in the second phase, while morphine, the standard drug, reduced licking time in both Phase 1 and Phase 2.

Phytotherapy, the medicinal use of plant‐derived compounds, has attracted substantial attention for its ability to alleviate a wide range of ailments affecting a significant portion of the global population.^[23]^ Notably, this therapeutic approach targets critical symptoms such as pain and oxidative stress, which are commonly associated with numerous chronic and acute conditions.^[24]^ Our study on *O. majorana* L. underlines the relevance of phytotherapy in this context. By examining the phytochemical composition and biological activities of *O. majorana*, we aim to elucidate its potential in mitigating these physiological challenges through its natural bioactive compounds.

The objective of this study was to evaluate the phytochemical composition, acute toxicity, antioxidant activity, and pharmacological effects of the aqueous extract of *O. majorana* L. (AEOM). The phytochemical analysis revealed significant amounts of total polyphenols, flavonoids, and condensed tannins, all known for their potential health benefits. AEOM exhibited notable antioxidant effects in both DPPH and reducing power tests. Additionally, the acute toxicity study revealed the safety of the extract up to a dose of 5000 mg/kg. HPLC analysis identified 11 secondary metabolites, with chlorogenic acid as the major component. The results of the pain tests demonstrated significant positive responses, thereby highlighting the effectiveness and promising potential of AEOM in modulating this complex physiological process.

Polyphenols are essential in determining how natural chemicals behave biologically. Phytochemical screening in this investigation indicated a significant amount of flavonoids, polyphenols and modest level of condensed tannins. We found numerous parallels and some discrepancies that may be attributed to both internal and extrinsic factors when we compared our results with those of other studies previously obtained for the same collected species but in various parts of the world.[^25^, ^26^, ^27^] Studies carried out in Morocco have shown that there are small differences in the amounts of condensed tannins, flavonoids, and polyphenols. Variations may be attributed to variations in the plant organ, habitat, genotype, and kind of solvent employed in the extraction procedure.^[28]^ Specifically, the effects of edaphic, climatic, and genetic factors.[^29^, ^30^]

The investigation employed various methods to evaluate the antioxidant properties of the aqueous extract. Several established assays, including the DPPH radical scavenging assay and the FRAP assay, were used to assess the antioxidant capacity of the extract. The findings indicated that AEOM exhibited significant antioxidant activity by efficiently scavenging free radicals and reducing oxidative damage, as evidenced by its effectiveness in neutralizing the DPPH radicals and enhancing the FRAP values. The results of the antioxidant activity of AEOM showed strong anti‐DPPH activity, with an IC_50_ of 0.3 mg/mL, as well as significant reducing and iron‐chelating activities.^[31]^ Similarly, the ethanolic extracts showed positive results in terms of antioxidant activity with IC_50_ values of 11.5 mg/mL for anti‐DPPH and 67.2 mg. Additionally, other studies, such as those conducted by Roby et al. (2013)^[1]^ and Dhull et al. (2016^[32]^ have also validated the antioxidant activity of *O. majorana* leaf extracts, demonstrating good activity in various testing techniques, such as the reduction of DPPH and ABTS free radicals^[33]^ These results highlight the strong antioxidant profile of oregano compounds and their medical importance.^[34]^

Additionally, the extract demonstrated a high total phenolic content, suggesting the presence of potent antioxidants in the plant material. As demonstrated by Nagendrappa,^[35]^ phenolic compounds may directly contribute to the antioxidative effects. Flavonoids, known for their robust antioxidant activities in laboratory settings, have the ability to scavenge various reactive oxygen species (ROS) such as superoxide and nitric oxide radicals.^[36]^ This investigation into the antioxidant potential of AEOM has uncovered compelling results, emphasizing its noteworthy antioxidant activity. The research aimed to explore the potential benefits of this extract in addressing oxidative stress, a factor known to contribute to various diseases and aging processes.^[37]^

The acute toxicity study showed that oral administration of AEOM at single doses (1000, 2000, and 5000 mg/kg body weight) did not result in mortality in mice; the animals survived until the end of the study. Similarly, the growth and relative organ weight of the AEOM‐treated animals remained unchanged throughout the experimental period. Biochemical analysis revealed no significant changes in renal and liver function parameters, while hematological analysis showed no effects on on the levels of white blood cell, red blood cell, and platelets. Histopathological examination revealed no morphological abnormalities in kidney, liver, and spleen tissues. Therefore, the LD_50_ of AEOM was higher than 5000 mg/kg. According to Kennedy Jr. et al.,^[38]^ substances with LD_50_ values greater than 5.0 g/kg when orally administered are generally considered practically non‐toxic. These findings are in line with previous studies demonstrating the safety of the aqueous extract of *O. majorana* aerial parts.^[39]^

The hotplate test is among the most common tests to evaluate the activity of opioid compounds, which are centrally acting analgesics in several animal species. This test is notable for its tendency to respond to painful stimuli that pass through neural pathways. It represents a supra‐spinal organized pain response.[^40^, ^41^] Based on our results, the aqueous extract of *O. majorana* showed anti‐nociceptive activity. These findings are in agreement with results of Fachini‐Queiroz et al.^[42]^ that demonstrated that luteolin, thymol, carvacrol, and ursolic acid in the *O. majorana* extract would be responsible for the anti‐nociceptive activity of this plant.^[43]^

On the other hand, the writhing test is applied to show peripheral anti‐nociceptive activity in mice. It is appropriate for differentiating between central and peripheral nociception.^[44]^ The injection of acetic acid produces peritoneal inflammation, which triggers a response characterized by cramps.^[45]^ Previous studies have shown that acetic acid indirectly induces the release of endogenous pain mediators (such as prostaglandins, kinins, histamine, etc.) that stimulate nociceptive neurons, which are sensitive to nonsteroidal anti‐inflammatory drugs and opioids.^[46]^ Thus, the aqueous extract of *O. majorana* showed a significant decrease in the number of writhes compared to the control group and the ASA‐treated group. *O. majorana*, commonly known as marjoram, exhibits significant analgesic effects, primarily attributed to its aqueous extract, whose major compounds are gallic acid, caffeic acid, and chlorogenic acid, according to several studies. The analgesic properties of *O. majorana* are linked to multiple mechanisms, including the modulation of cholinergic, opioid, and dopaminergic systems, as well as the inhibition of N‐Methyl‐D‐aspartate (NMDA) receptors and the reduction of nitric oxide (NO) production.^[47]^ These pathways contribute to the antinociceptive effects of these compounds, which correspond to the action of traditional non‐steroidal anti‐inflammatory drugs (NSAIDs) that inhibit the prostaglandin metabolic pathway, as noted by Okazaki et al.^[48]^ who reported the antiplatelet activity of the methanol extract of O. majorana. Furthermore, the ability of *O. majorana* to modulate inflammatory responses by inhibiting pro‐inflammatory cytokines, such as IL‐1β, IL‐6, interferon (IFN)‐α, and TNF‐α, which are key players in the pathways of fever and pain, highlights its potential therapeutic applications in pain and inflammation management. The richness in polyphenols and flavonoids in the aqueous extract of *O. majorana* not only enhances its analgesic effects but also underscores the complex interaction of the plant with the mechanisms of the central nervous system, offering a promising natural alternative for pain relief.

## Conclusions

Our investigation into the therapeutic potential of the *Origanum majorana* L. extract underscores the promising role of herbal medicine in addressing diverse health challenges, particularly those related to pain management and oxidative stress. Through rigorous analyses including phytochemical composition, acute toxicity evaluation, antioxidant activity, and pharmacological effects, we have revealed compelling insights. The abundant presence of polyphenols, flavonoids, and condensed tannins in the extract, as well as its robust antioxidant effects, indicate its potential in combating oxidative damage. Safety assessments up to a dose of 5000 mg/kg in acute toxicity studies align with previous safety profiles of similar extracts, affirming its suitability for therapeutic use. HPLC analysis identified key secondary metabolites like chlorogenic acid contributing to its bioactivity. Notably, our pain tests demonstrated significant anti‐nociceptive and peripheral analgesic activities, suggesting a promising avenue for pain management. These findings not only contribute to the growing body of evidence supporting the therapeutic efficacy of *O. majorana* L. extract but also highlight its potential applications in holistic healthcare approaches, paving the way for further research and clinical exploration in phytotherapy.

## Experimental Section

### Plant Samples

The leaves of *O. majorana* were collected in 2022 from Azilal, a region in the central Atlas Mountains of Morocco (31°57′41′′ N, 6°34′15′′ W). The plant's authenticity was initially confirmed by botanist Professor A. Ouhammou and pharmacologist Professor A. Chait from the Faculty of Sciences at Semlalia, Cadi Ayyad University. The plants were then carefully preserved and recorded under voucher specimen MARK‐14381 in the herbarium of the Department of Biology, Faculty of Sciences Semlalia, Cadi Ayyad University, Marrakech, Morocco.

### Extract Preparation

The process began with macerating the leaves of *O. majorana* in water, with continuous agitation for 12 hours. Afterward, the resulting liquid underwent centrifugation at 1200 rpm, followed by filtration and lyophilization using a Christ instrument. The resulting dry powder was then aseptically stored in amber bottles at 4 °C until needed for further use.

### Phytochemical Study

#### Total Phenolic Content (TPC)

The total phenolic content (TPC) of the extract was measured using a modified Folin‐Ciocalteu method based on Singleton et al..^[49]^ To begin the assessment, 0.4 mL of the diluted extract was mixed with 1.5 mL of Folin‐Ciocalteu reagent. Afterward, 1.6 mL of a 7.5 % sodium carbonate solution was added. The mixture was then kept in the dark at room temperature for 2 hours, and the absorbance was read at 765 nm. Gallic acid was used as the standard reference, and the results were expressed in milligrams of gallic acid equivalents per gram of dry weight (mg GAE/g DW).

#### Total Flavonoid Content (TFC)

The total flavonoid content (TFC) of the extract was determined following the method outlined by Zhishen et al..^[50]^ Initially, 200 μL of the extract was mixed with 1 mL of distilled water. Subsequently, 60 μL of 5 % NaNO_2_ and 60 μL of 10 % AlCl_3_ were introduced into the solution. After a five‐minute incubation period, 400 μL of 1 M NaOH was added, and the absorbance was measured at 510 nm. The TFC was then expressed as milligrams of catechin equivalents per gram of dry matter (mg CE/g DM).

#### Total Condensed Tannins

To assess the condensed tannin content, the method outlined by Aitbaba et al.^[51]^ was followed. In summary, 400 μL of the diluted samples were combined with 3 mL of a 4 % methanol vanillin solution and 1.5 mL of concentrated HCl. Following a 15 minute incubation period, the absorbance was measured at 500 nm. The condensed tannin content was then determined and expressed as milligrams of catechin equivalents per gram of dry matter (mg CE/g DM).

### Antioxidant Activity

#### Radical Scavenging Activity (DPPH)

The antioxidant potential of the extract was assessed using the 2,2‐diphenyl‐1‐picrylhydrazyl (DPPH) assay, following the method described by Mansouri et al.^[52]^ with modifications from Baslam et al..^[53]^ In brief, 1.5 mL of a methanolic DPPH solution (6×10^−5^ M) was mixed with 60 μL of the extract at various concentrations (1, 2, 4, 6, and 8 mg/mL) of AEOM. The mixture was protected from light and left at room temperature for 30 minutes. After the incubation period, the absorbance was measured at 515 nm. A negative control was prepared by mixing 1.5 mL of the DPPH solution with 60 μL of methanol. Positive controls consisted of butylated hydroxytoluene (BHT) and quercetin. The percentage of inhibition was determined using the following formula:

%Inhibition=[(Acontrol-Asample)/Acontrol]×100

Where A control represents the absorbance of the control, and A sample denotes the absorbance of the test compound.

The concentration of the sample that causes 50 % inhibition (IC_50_) was ascertained from a graph illustrating the percentages of inhibition plotted against the sample concentrations.

#### Ferric Reducing Ability Power (FRAP)

The FRAP test, as outlined by Oyaizu,^[54]^ evaluates the inhibition of Fe (II)‐Ferrazine complex formation during sample incubation with ferrous iron. In detail, a mixture of 1 mL distilled water, 2.5 mL phosphate buffer (0.2 M, pH 6.6), and 2.5 mL potassium ferricyanide (K_3_[Fe(CN)_6_], 1 %) was combined with 0.5 mL of extract solutions at varying concentrations. Following a 30 minute incubation, 2.5 mL distilled water, 2.5 mL 10 %trichloroacetic acid, and 0.5 mL FeCl_3_ were introduced into the mixture. Absorbance was then measured at 700 nm. Quercetin and butylated hydroxytoluene (BHT) served as positive controls.

### Animal Study

#### Animals

Adult male Swiss mice (25–35 g) were obtained from the animal care unit of the Faculty of Science Semlalia, Cadi Ayyad University, Marrakech, Morocco. These animals were housed under controlled conditions at a constant room temperature (22±2 °C) with a 12 hour light/12 hour dark cycle and had unrestricted access to food and water. All animal procedures strictly complied with the guidelines outlined in the European Council Directive (EU2010/63). The study was conducted ethically and received approval from the Council Committee of the Research Laboratory, Faculty of Science, Cadi Ayyad University of Marrakech. Every effort was made to minimize potential animal suffering.

#### Acute Toxicity

The limit test dose for the acute toxicity study (5000 mg/kg) was conducted in accordance with the guidelines of the Organization for Economic Cooperation and Development (OECD) (guideline n. 423). Four groups of mice were used, each comprising six animals. Three groups received oral administration of AEOM at doses of 1000, 2000, and 5000 mg/kg, respectively, while the remaining group (negative control) received distilled water. Administration was performed at a rate of 10 mL/kg. The mice were closely monitored for signs of toxicity and mortality during the first two hours following the administration of the extract.

#### Biochemical and Histological Analyses

On day 14, the animals underwent euthanasia via cervical dislocation to obtain blood samples for the assessment of potential changes in hematological and biochemical parameters. Organs, including the liver, spleen, and kidneys, were preserved in buffered formalin (10 %) and subsequently embedded in paraffin. Following this, 5 μm sections were obtained using a microtome and subjected to hematoxylin and eosin staining for microscopic examination.

### Analgesic Tests

#### Hot Plate Test

The methodology previously employed by Okolo et al.^[55]^ was implemented in this study. The animals were subjected to a test environment comprising a glass cylinder placed on a heated metal plate maintained at a temperature of 55±1 °C. The reaction time was gauged by measuring the latency to unpleasant responses such as licking, shaking one paw, and jumping. The treated mice received three oral doses of AEOM (200, 400, and 800 mg/kg), while the control group was orally administered 10 mL/kg of water. An intraperitoneal injection of 10 mg/kg of morphine was also administered. After treatment, latencies to nociceptive responses were measured at intervals of 30, 60, 90, and 120 minutes.

#### Abdominal Writhing Induced by Acetic Acid

The procedure outlined by Koster et al., 1959^[43]^ was employed in this study. In summary, intra‐peritoneal injections of 0.6 % acetic acid (0.1 mL/10 g) were administered to mice. Thirty minutes before the injections, all experimental groups (5 mice per group) were treated with either the vehicle, aspirin (200 mg/kg), or the tree doses of the AEOM. Subsequently, individual cages were designated for each group to facilitate counting of abdominal spasms over a 30 minute period.

#### Paw Licking Induced by Formalin

The experiment adhered to the methodology described by Hunskaar & Hole,.^[56]^ Mouse groups (n=5) were orally administered various doses of AEOM (250, 500, and 1000 mg/kg) 45 minutes before receiving a 20 μL injection of 2 % formalin (v/v in 0.9 % saline) into the subplantar area of the right hind paw. The control group received a vehicle (10 mL/kg of saline). The reference analgesic medications, morphine (10 mg/kg, i. p.) and aspirin (200 mg/kg, i. p.), were included. Following formalin injection in each mouse, the duration of paw licking was measured during two intervals: 0–5 min (neurogenic phase) and 15–30 min (inflammatory phase).

### Statistical Analysis

Data from each measurement were presented as mean values±SEM (standard error of the mean). The analysis and presentation of results were conducted using SigmaPlot 12.0 software, employing one‐way analysis of variance (one‐way ANOVA). In cases where differences were deemed significant (*p*<0.05), post hoc analysis techniques were applied.

## Funding

This study did not receive financial support.

## Ethical Issue

Reducing the number of animals and suffering were done according to the recommendations of the European Council Directive (EU2010/63), and authorized by the council committee of the faculty of Sciences, Cadi Ayyad University Marrakech Morocco.

## Author Contributions

M.A.El Amiri: Conceptualization, Data curation, Formal analysis, Writing‐original draft preparation, Validation; H. Kabdy: Data curation, Validation, Writing‐original draft preparation; A. Aitbaba: Data curation, Writing‐original draft preparation; J. Laadraoui: Formal analysis, Validation; A. Baslam: Formal analysis, Validation; R. Aboufatima: Conceptualization; L. El Yazouli: Formal analysis, Validation; S. Moubtakir: Formal analysis, Validation S. Garzoli: Writing‐review and editing, Supervision; A. Chait: Conceptualization, Writing‐review and editing, Supervision.

## Conflict of Interests

Authors declare no conflict of interest.

1

## Data Availability

The data that support the findings of this study are available from the corresponding author upon reasonable request.
